# Genetic Characterization of Human-Derived Hydatid Cysts of *Echinococcus granulosus* Sensu Lato in Heilongjiang Province and the First Report of G7 Genotype of *E. canadensis* in Humans in China

**DOI:** 10.1371/journal.pone.0109059

**Published:** 2014-10-16

**Authors:** Tiemin Zhang, Dong Yang, Zhaolin Zeng, Wei Zhao, Aiqin Liu, Daxun Piao, Tao Jiang, Jianping Cao, Yujuan Shen, Hua Liu, Weizhe Zhang

**Affiliations:** 1 Department of General Surgery, the First Affiliated Hospital of Harbin Medical University, Harbin, Heilongjiang, China; 2 Department of Parasitology, Harbin Medical University, Harbin, Heilongjiang, China; 3 Department of General Surgery, the Second Affiliated Hospital of Harbin Medical University, Harbin, Heilongjiang, China; 4 National Institute of Parasitic Diseases, Chinese Center for Disease Control and Prevention, Key Laboratory of Parasite and Vector Biology, Ministry of Health, WHO Collaborating Centre for Malaria, Schistosomiasis and Filariasis, Shanghai, China; Oxford University, United Kingdom

## Abstract

Cystic echinococcosis (CE) caused by the larval stage of *Echinococcus granulosus* sensu lato (s.l.) is one of the most important zoonotic parasitic diseases worldwide and 10 genotypes (G1–G10) have been reported. In China, almost all the epidemiological and genotyping studies of *E. granulosus s.l.* are from the west and northwest pasturing areas. However, in Heilongjiang Province of northeastern China, no molecular information is available on *E. granulosus s.l.* To understand and to speculate on possible transmission patterns of *E. granulosus s.l.*, we molecularly identified and genotyped 10 hydatid cysts from hepatic CE patients in Heilongjiang Province based on mitochondrial cytochrome c oxidase subunit I (*cox1*), cytochrome b (*cytb*) and NADH dehydrogenase subunit 1 (*nad1*) genes. Two genotypes were identified, G1 genotype (n = 6) and G7 genotype (n = 4). All the six G1 genotype isolates were identical to each other at the *cox1* locus; three and two different sequences were obtained at the *cytb* and *nad1* loci, respectively, with two *cytb* gene sequences not being described previously. G7 genotype isolates were identical to each other at the *cox1*, *cytb* and *nad1* loci; however, the *cytb* gene sequence was not described previously. This is the first report of G7 genotype in humans in China. Three new *cytb* gene sequences from G1 and G7 genotypes might reflect endemic genetic characterizations. Pigs might be the main intermediate hosts of G7 genotype in our investigated area by homology analysis. The results will aid in making more effective control strategies for the prevention of transmission of *E. granulosus* s.l.

## Introduction

Cystic echinococcosis (CE, also known as hydatidosis or hydatid disease) caused by the larval stage of *Echinococcus granulosus* sensu lato (s.l.) is considered as one of the most important zoonotic parasitic diseases worldwide, particularly affecting pastoral and poor rural communities where people raise livestock in close contact with dogs [1]. The disease causes great economic loss in livestock through condemnation of infected organs (mainly liver). More importantly, CE can be responsible for a life-threatening infection in humans. Fatal cases of human CE have been reported with inoperable CE cysts of the brain and anaphylactic shock caused by rupture of liver hydatid cysts [2], [3].

Humans become infected with CE by ingesting eggs released from dogs or other canids through direct contact with the animals and consumption of food and water contaminated with infected animal feces. Eggs hatch in small intestine and parasite larvae (hydatid cysts) can be found in almost any organ, with the liver being the most common. Usually, CE may develop silently over years and even decades until it surfaces with clinical signs or symptoms. Clinical symptoms are mainly related to the localization, size, and number of cysts. However, a recent study described that there seemed to be a relationship between the genotypes and the size of hydatid cysts, in which all the patients infected with G7 genotype showed smaller liver cysts than those infected with G1 genotype [3].

Molecular epidemiological data have identified 10 genotypes (G1–G10) within *E. granulosus s.l.* In China, to date, three genotypes have been found, G1, G3 and G6 genotypes in humans and G1 and G6 genotypes in animals, with G1 genotype being predominant in both of them [4]–[20] (Table 1). Currently, G1–G3, G4, G5 and G6–10 genotypes are designated to *E. granulosus* sensu stricto (s.s.), *E. equinus*, *E. ortleppi* and *E. canadensis*, respectively [1], [21].

**Table 1:** Genotypes of *E. granulosus s.l.* in humans and animals in China.

Province	Human				Animal			Ref.
	No. ofSamples	Genotype			No. ofsamples	Genotype (host)		
		G1	G3	G6		G1	G6	
Gansu	1	1			12	12 (sheep)		[4], [5]
Ningxia	12	12			16	14 (sheep),1 (goat), 1(squirrel)		[5], [6]
Qinghai	38	38			178	122 (sheep),29 (yak),21 (cattle),4 (dog)	2 (goat)	[4], [5], [7]–[11]
Qinghaiand Sichuan	70*	70			76*	57 (sheep),19 (yak)		[12], [13]
Sichuan	54	52	2		34	10 (sheep),24 (yak)		[5], [7], [10], [11], [14], [15]
Tibet					34	30 (sheep),2 (yak)	2 (sheep)	[7], [10], [11]
Xinjiang	149	145		4	134	63 (sheep),5 (cattle),1 (camel),59 (dog)	2 (cattle),1 (camel),3 (dog)	[4], [13], [16]–[20]

*No specific description on geographical source of hydatid cysts in the two provinces.

CE is one of the major parasitic problems in humans in China. Human CE cases have been reported in 27 provinces, autonomous regions, and municipalities, with western and northwestern China being the main endemic areas [22], [23]. Although Heilongjiang Province in the northeast of China is not a main endemic area of CE, the number of CE patients in hospitals has been increasing, especially in recent years since the first human CE case was reported in 1958 [24]. Dogs, pigs and sheep have been confirmed to be infected with *E. granulosus s.l.* [25], [26]. In fact, pigs, sheep and cattle are the main economic animals, and domestic dogs are kept in large numbers by local inhabitants to guard property and livestock with the development of livestock husbandry. Thus, people have a large risk of CE infection by farming activities and home-slaughtering practices. Unhealthy life habits also increase the possibility of human infection by these pathogens via eating uncooked vegetables and drinking raw water. However, no genotyping data are available about *E. granulosus s.l.* isolates in this area.

Mitochondrial DNA (mtDNA) is reported to be more powerful than nuclear DNA within *E. granulosus s.l.* in constructing phylogenetic relationships among closely related species because of its rapid sequence evolution [27]. Large datasets derived from mitochondrial genomes also have the potential to resolve problematic issues in *Echinococcus* taxonomy [13], [28]. The aims of the present study were to molecularly identify and genotype hydatid cysts of CE patients residing in Heilongjiang Province by sequencing and analyzing mitochondrial cytochrome c oxidase subunit I (*cox1*), cytochrome b (*cytb*) and NADH dehydrogenase subunit 1 (*nad1)* genes, to speculate on possible transmission patterns of this cestode by homology analysis, and to understand the phylogeny of genotypes of *E. granulosus s.l.* by constructing neighbor-joining trees at three loci above. The results will contribute to developing control strategies of CE in our area.

## Materials and Methods

### Ethics statement

This research study was approved by the Medical Ethics Review Committee of Harbin Medical University. All adult subjects gave their written informed consent for surgery. Medical Ethics Review Committee of Harbin Medical University exempted the individual informed consent for molecular identification of hydatid cysts because samples (hydatid cysts) were obtained post surgically and CE patients’ names have not appeared in this study.

### Collection of hydatid cyst samples

A total of 10 hydatid cysts were collected from 10 patients suffering from hepatic CE while the size of the cysts was recorded, ranging from 6.8 cm to 10.1 cm. All the patients came from Heilongjiang Province and were operated surgically at the First and the Second Affiliated Hospital of Harbin Medical University and during May 2013 to December 2013. The endocysts were preserved in 70% ethanol at 4°C.

### Microscopic examination of protoscoleces

To confirm and obtain the protoscoleces of cyst samples, suspension liquid of a part of endocysts rinsed by physiological saline was centrifuged at 1500 g for five min at room temperature, and a wet preparation was made from the sediment. The slides were examined microscopically at 100× and 400× magnification.

### Extraction of genome DNA

Genomic DNA was extracted from approximately 25 mg of sediment positive for protoscoleces using the DNeasy blood and tissue kit (Qiagen, Germany) according to manufacturer’s instructions. DNA was eluted in 200 µL of Buffer AE and was stored at –20°C until further use in PCR analysis.

### Primers and cycle parameters of PCR amplification

The mitochondrial *cox1*, *cytb* and *nad1* genes of hydatid cysts were amplified using previously published primers. Approximately 800 bp of the *cox1* gene and 580 bp of the *cytb* gene were amplified, with the same cycle parameters, as follows: 35 cycles at 94°C for 30 s, 54°C for 30 s, and 72°C for 60 s [14]. Approximately 900 bp of the *nad1* gene was amplified using the following primers: 5′-GGGCTATTCTCAGT(C/T)TCGTA-3′ and 5′-ACCCAAAACCCACATTCT(G/T)-3′, with the cycle parameters as follows: an initial denaturation step at 95°C for 3 min; 30 cycles at 94°C for 1 min, 50°C for 50 s, and 72°C for 70 s, followed by a final extension step at 72°C for 10 min [4].

Each DNA preparation was analyzed at least three times by PCR. All PCR amplifications were run with negative control (non-template water control). After agarose gel electrophoresis (1.5%), all PCR products of *cox1*, *cytb* and *nad1* genes were purified and sequenced directly to identify genotypes of *E. granulosus s.l.*

### DNA sequencing and molecular analysis

All PCR products were sequenced with PCR primers at each locus on an ABI PRISMTM 3730 DNA Analyzer (Applied Biosystems, Carlsbad, CA, USA), using a BigDye Terminator v3.1 Cycle Sequencing kit (Applied Biosystems, USA). Accuracy of the sequencing data was confirmed by sequencing in both directions. Nucleotide sequences obtained in the present study were subjected to BLAST searches (http://www.ncbi.nlm.nih.gov/blast/), and were then aligned and analyzed with each other and *E. granulosus* reference sequences downloaded from GenBank using Clustal X 1.83.

### Phylogenetic analysis

To understand phylogeny of genotypes of *E. granulosus s.l.*, three neighboring-joining trees were constructed at *cox1*, *cytb* and *nad1* loci, respectively, using the software Mega 5 (http://www.megasoftware.net/) based on the evolutionary distances calculated by Kimura-2-parameter model. The reliability of these trees was assessed using the bootstrap analysis with 1000 replicates.

### Nucleotide sequence accession numbers

Representative nucleotide sequences obtained in this study were deposited in the GenBank database under the accession numbers KJ556989 to KJ556997.

## Results

All the cysts were confirmed to be fertile (with protoscoleces) by microscopy. Two genotypes were identified out of 10 hydatid cysts based on the *cox1*, *cytb* and *nad1* genes: G1 (n = 6) and G7 (n = 4). However, one cyst identified as G7 genotype was not amplified successfully at the *cytb* locus.

### Genetic characterizations of G1 genotype of *E. granulosus s.s.*

Six *cox1* gene sequences identified as G1 genotype were identical to each other (KJ556996) and showed 100% similarity with those from humans in Mongolia (AB787540–45, AB787547), Russia (AB777904, AB777907 and AB777908), and China (AB688608, AB688616, AB688617, AF458874 and JF906154); from sheep in Jordan (AB688591, AB688594), Iran (JQ250806), Peru (AB688621) and China (EU072107, AY377836 and AB491414); from cattle (JQ318001 and AY278068) and yaks (JF906154) in China; from a cat in Russia (AB622277).

Three different *cytb* gene sequences were obtained from six hydatid cysts identified as G1 genotype, with one to three base differences between one another. One sequence (KJ556990) (n = 4) had 100% similarity with the human-derived (AB786664) and yak-derived (AY278067) sequences in China, and a cat-derived sequence (AB622276) in Russia. The other two (KJ556991 and KJ556992) were not described previously, having 99.83% and 99.65% similarity with that from a cat (AB622276), respectively. However, only the base difference (A to G) led to change of one amino acid (isoleucine to valine) (Table 2).

**Table 2:** Difference in bases and amino acids at the *cytb* locus of *E. granulosus s.l.*

Genotype/species	Accession no.	Homology (%)	Codon (amino acid)/Nucleotide position a		
G1/*E. granulosus* s.s.	AB622276	Ref sequence	ATT (I)/520	TAC (Y)/612	TTG (L)/721
	KJ556990	100	ATT (I)/520	TAC (Y)/612	TTG (L)/721
	KJ556991	99.83	ATT (I)/520	TAT (Y)/612	TTG (L)/721
	KJ556992	99.65	GTT (V)/520	TAC (Y)/612	CTG (L)/721
G7/*E. canadensis*	AB235847	Ref sequence	ATT (I)/451	TTA (L)/855	
	KJ556989	99.65	GTT (V)/451	TTG (L)/855	

a Nucleotide position numbers according to AB235847, with the beginning of the coding region of the *cytb* gene as position no. 1.

Two different *nad1* gene sequences were obtained from six hydatid cysts identified as G1 genotype, and there was one base difference between them. One sequence (KJ556993) (n = 5) had 100% similarity with the sequences from humans in China (JX217926); from sheep in China (JX217856, JX217868, JX217883 and JX217895), Morocco (EF367295–EF367301) and Iran (FJ010971–FJ010973 and GQ358009–CQ358013); from goats in Iran (HM055621–HM055624 and GQ358008); from cattle in Iran (HM055617 and GQ358005); from a camel in Iran (GQ358001); from a yak in China (JX217911). The other one (KJ556994) had 100% homology with the sequence from a human in China (JX217920).

### Genetic characterizations of G7 genotype of *E. canadensis*

Four *cox1* gene sequences identified as G7 genotype were identical to each other (KJ556997) and showed 100% similarity with those from a human in Argentina (JN176935) and from pigs in Peru (AB777924, AB777925 and AB458678), Argentina (GU980914) and Romania (EU048820).

Three *cytb* gene sequences were obtained in the present study. All of them were identical to each other and were not described previously. The representative *cytb* gene sequence (KJ556989) had 99.65% similarity with that from a pig (AB235847), having two base variations. However, only one base variation (A to G) led to change of one amino acid (isoleucine to valine) (Table 2).

Four *nad1* gene sequences of G7 genotype were identical to each other (KJ556995) and were completely homologous to pig-derived sequences in Slovakia (AJ241189) and Poland (AJ241196 and AJ241212).

## Discussion

Human CE is a cause of serious concern due to increase in morbidity in humans and economic impact of diagnosis and treatment in China; however, the degree of endemicity of echinococcosis differs with regions in China [22], [23]. The fact of increasing number of sporadic CE patients in Heilongjiang Province arouses our interest in understanding genetic characterization of *E. granulosus* isolates, which will be helpful to speculate on possible transmission patterns.

In the present study, six of ten hydatid cysts were identified as G1 genotype of *E. granulosus s.s.* G1 genotype is reported to have a predominance in humans and animals in most studies [23]. Molecular epidemiological data of CE have revealed that G1 genotype is responsible for 88.5% of human CE worldwide [1]. In China, G1 genotype can be seen in CE cases, 98.1% (318/324) for humans versus 97.9% (474/484) for animals (Table 1). Meanwhile, differences in the genetic characterizations within G1 genotype strains were noticed in the present study. Six *cox1* gene sequences were identical to each other while three and two different gene sequences were obtained at the *cytb* and *nad1* loci, respectively, showing a conservative nature of G1 genotype at the *cox1* locus. However, in Algeria, eight and three different gene sequences of G1 genotype were obtained among 30 hydatid cysts from humans, cattle and sheep at the *cox1* and *nad1* loci, respectively [29]. In Kenya, 49 hydatid cysts of G1 genotype from humans had 100% homology with each other at the *cox1* and *nad1* loci, respectively [30]. In addition, the findings of the two new *cytb* gene sequences of G1 genotype (KJ556991 and KJ556992) also possibly reflected endemic genetic characterizations of *E. granulosus s.l*. However, the base variation of the *cytb* sequence (KJ556991) was found to be insignificant due to the degeneracy of amino acids, for the two codons (TAC and TAT) can be translated to the same amino acid (tyrosine) (Table 2).

In the present study, four hydatid cysts were identified as G7 genotype, which currently has been assigned to *E. canadensis*, together with G6, G8 and G10 genotypes. To date, G7 genotype has been reported in humans from seven countries [3], [31]–[34] and in five animal species as intermediate hosts from 12 countries [32], [34]–[54] (Table 3). In general, G7 genotype is not as common as G1 genotype either in humans or in animals worldwide. However, it has been reported that 100% (30/30), 92.0% (23/25) and, 33.3% (9/27) of human-derived hydatid cysts were indentified as G7 genotype in Poland, Austria and the former Yugoslavia, respectively [3], [31]. Differences in population of *E. granulosus s.l.* might be related to geographical locations. Homology analysis revealed that four hydatid cysts had 100% similarity with those from pigs [39], [47] at the *cox1* and *nad1* loci (AJ241189, AJ241196 and AJ241212). The result showed that pigs might be intermediate hosts of G7 genotype of *E. canadensis* in Heilongjiang Province. In fact, early in 2000, 4.5% of pigs were reported to be infected with hydatid cysts in our province [25]. Based on our summary of previous molecular data of *E. granulosus s.l.*, pigs appear to be a principal animal intermediate host for G7 genotype, accounting for the highest prevalence of 82.8% (173/209) in pigs (Table 3). In Heilongjiang Province, the pig industry is a major economic component. Once pigs are infected with *E. granulosus s.l.*, the habit of feeding raw offal of animals to dogs will easily lead to *E. granulosus s.l.* infection in dogs. Thus, this parasite can complete its life cycle in our area.

**Table 3:** Distribution of G7 genotype of *E. canadensis* in humans and animals worldwide.

Country	No. ofhumans	No. ofanimals (host)	Amplifiedgene(s)	Ref.
Argentina		76 (pig)	*cox1*, *nad1*, ITS1	[35]–[40]
Austria	23		*nad1*	[3]
Brazil		3 (pig),1 (cattle)	*cox1*, *mdh*, EgAgB4	[41], [42]
FormerYugoslavia	9		*nad1*	[3]
Greece		20 (goat)	*cox1*, *nad1*	[43]
Hungary	1		*nad1*	[3]
Italy		2 (pig)	*cox1*, *nad1*,12 S rRNA	[44]
Mexico		8 (pig)	*cox1*, *nad1*,ITS1, Eg9, Eg16	[45], [46]
Peru		12 (pig)	*cox1*, *nad1*, *ef1a*	[47], [48]
Poland	30	2 (pig)	*cox1*, *nad1*, ITS1	[31], [49]
Portugal		1 (cattle)	*cox1*	[50]
Romania		18 (pig)	*cox1*, *nad1*,*mdh*, EgAgB4	[41], [51]
Slovakia	2	18 (pig),2 (cattle)	*nad1*, ITS1,12S rRNA	[32], [52]
Spain		34 (pig),4 (wild boar),6 (goat)	*cox1*, *nad1*	[53], [54]
South Africa	2		*nad1*, 12S rRNA	[33]
Turkey	1	2 (sheep)	*cox1*, *nad1*, 12S rRNA	[34]

This is the first report of G7 genotype in humans in China. Identification of G7 genotype might also be attributable to the fact of the increasing international travels and livestock trades as well as the increasing number of dogs imported in recent decades. Of course, we could not rule out the possibility that humans affect CE by contacting local dogs. Early in 2002, 4% dogs were reported to be infected with *E. granulosus s.l.* in Heilongjiang Province [25]. However, they were only identified by microscopy. Thus, true transmission patterns of CE in investigated area still need to be elucidated by molecularly analyzing more samples from animals in the future.

**Figure 1:**
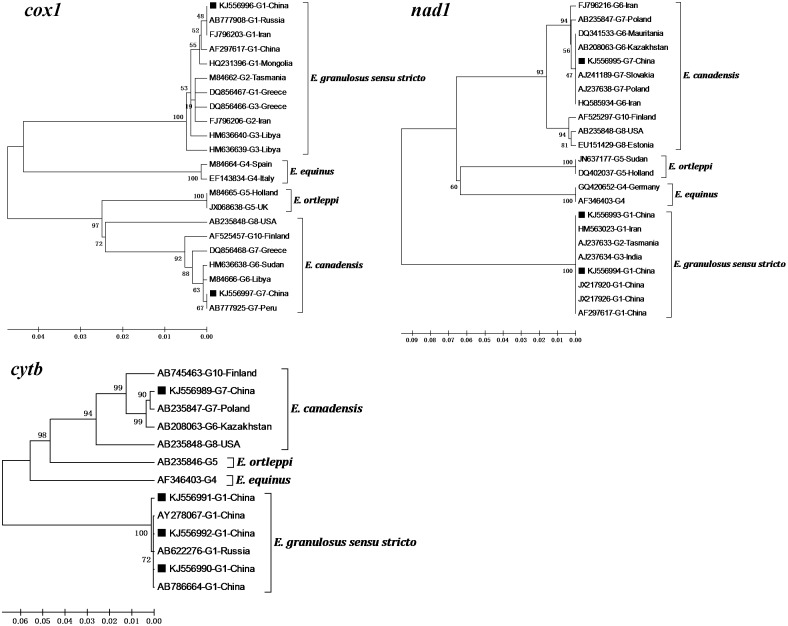
Comparison of Phylogenies of different genotypes within *E. granulosus s. l.* based on *cox1, cytb* and *nad1* sequences. Three phylogenetic trees were constructed using the Neighbor-joining distance method analysis with a Kimura-2-parameter model. The reliability of these trees was assessed using the bootstrap analysis with 1000 replicates. The GenBank accession number, the genotype and the country of origin were given for each isolate of *E. granulosus s.l.* when available. The squares indicate the sequences of *E. granulosus s.l.* from the present study.

A single locus has been used in most of epidemiological studies of *E. granulosus s.l.* However, genetic data of several genes provide more information, which will be helpful to understand the taxonomic status and phylogeny between inter- and intra-genotypes of *E. granulosus s.l*., and are especially valuable to designate a new genotype/species of *E. granulosus s.l.* McManus questioned the validity of G9 genotype, since the three same *E. granulosus s.l.* isolates from humans were identified as G9 and G7 genotypes based on ITS1-RFLP and based on sequencing of the *nad1* gene [55], [56]. In the present study, by a phylogenetic analysis based on three neighbor-joining trees at the *cox1*, *cytb* and *nad1* loci, respectively (Fig. 1), it could be seen that at the *cox1* and *nad1* loci, it was impossible to separate G6 and G7 genotypes within *E. canadensis* as well as G1, G2 and G3 genotypes within *E. granulosus s.s*. However, the phenomenon that G6 and G7 genotypes of *E. canadensis* seemed to be different from each other at the *cytb* locus might be related to fewer sequences available currently. In spite of this, it is generally scientific and reasonable to regard G6 and G7 genotypes as one unit within *E. canadensis.*

Hydatid cysts of G1 genotype were observed to be a little larger than those of G7 genotype in the present study, with 9.7 cm versus 7.1 cm in diameter on average. A similar result was seen by comparing CE cysts from 68 patients infected with G1 genotype to those from 33 patients infected with G7 genotype conducted in Austria (10.7 cm for G1 genotype cysts and 5.9 cm for G7 genotype cysts) [3]. However, due to few data involving genotypes and sizes of hydatid cysts, it is unclear about the relationship between them.

To the best of our knowledge, the finding of G7 genotype (*E. canadensis* G6/7) in China shows that this genotype possibly has a wider geographical distribution than previously considered [57]. The previous reports of adults of *E. granulosus s.l.* in dogs, and hydatid cysts in sheep and pigs in Heilongjiang Province suggest the presence of two transmission patterns in our investigated areas: dog-sheep and dog-pig life cycles. However, the relationship between transmission patterns and species/genotype of *E. granulosus s.l.* needs to be elucidated by molecular epidemiological investigations from animals. We will try to seek the cooperation of CE patients to carry out a study of risk factors by questionnaire of their life style habits. The data will also be valuable to make control strategies for the prevention of transmission of this parasite.
